# From passive monitoring to active engagement: a systematic review and meta-analysis of digital health technologies for improving objective physical activity and cardiorespiratory fitness in patients with obstructive sleep apnea

**DOI:** 10.3389/fpubh.2026.1760571

**Published:** 2026-02-27

**Authors:** Jingkang Xia, Jiayi Yao, Haozhe Wang, Yongliang Zhu, Lihong Mao, Xueqiang Zhu

**Affiliations:** 1 School of Competitive Sport, Shandong Sport University, Rizhao, China; 2 School of Physical Education, China University of Mining and Technology, Xuzhou, China

**Keywords:** digital health interventions, meta-analysis, objective physical activity, obstructive sleep apnea, randomized controlled trials, systematic review, wearable devices

## Abstract

**Background:**

Physical inactivity is a critical factor exacerbating obstructive sleep apnea (OSA) and its associated comorbidities. Previous evidence has largely relied on subjective questionnaires, leading to a lack of definitive evidence based on objective measurements. This study aimed to systematically evaluate the effects of digital health interventions (DHIs) on objective physical activity, body composition, and sleep parameters in OSA patients, while further exploring the efficacy differences between active intervention and passive monitoring modes.

**Methods:**

A systematic search was conducted across five major databases (including PubMed and Embase) and registration platforms up to November 19, 2025. Only randomized controlled trials (RCTs) that assessed physical activity using objective tools were included. Bias was evaluated using the Cochrane Risk of Bias Tool 2.0 (RoB 2.0), and the quality of evidence was graded using the GRADE system. Statistical analyses utilized random-effects models to calculate Mean Differences (MD) and 95% Confidence Intervals (CI). Subgroup analyses were performed based on a refined classification of intervention modes, accounting for interaction patterns, theoretical foundations, and the application of behavior change techniques (BCTs).

**Results:**

A total of seven randomized controlled trials involving 693 patients were included. Overall meta-analyses revealed no statistically significant differences between digital interventions and standard care in increasing daily steps (MD = 86.94, *P* = 0.85), reducing body mass index (*P* = 0.74), improving the Apnea-Hypopnea Index (*P* = 0.31), or alleviating somnolence (*P* = 0.15). However, telerehabilitation-based interventions significantly improved peak oxygen uptake [MD = 342.41 ml/min, 95% CI (38.05, 646.77)]. Exploratory subgroup analyses and individual study data suggested that active intervention modes characterized by synchronous interaction or theory-driven protocols showed a greater tendency for weight loss compared to passive monitoring and significantly increased absolute step counts during the intervention period (*P* = 0.02). The overall quality of evidence for the primary outcomes was rated as low to moderate.

**Conclusion:**

Current evidence suggests that the application of digital health technology alone may be insufficient to significantly alter objective step counts or clinical outcomes in OSA patients, which might be largely attributed to the prevalence of passive monitoring modes lacking deep interaction in current studies14. Nevertheless, the physiological benefits observed in VO^2^max reveal the unique value of these interventions, and active modes integrating closed-loop feedback show potential in driving behavioral changes compared to passive monitoring. Future clinical strategies should shift toward theory-driven active interaction modes that integrate core BCTs, potentially providing a new pathway to further improve long-term cardiovascular prognosis beyond traditional treatments.

**Systematic review registration:**

[www.crd.york.ac.uk/prospero], identifier [CRD420251246601].

## 1 Introduction

Obstructive sleep apnea (OSA) has emerged as a global public health crisis, estimated to affect nearly 1 billion adults worldwide, and is closely associated with a significantly increased risk of hypertension, cardiovascular disease, and metabolic disorders (1, 2). Beyond typical symptoms such as daytime sleepiness, OSA patients frequently present with impaired cardiorespiratory fitness (CRF), characterized by reduced peak oxygen consumption (VO^2^peak) (3), which serves as an independent predictor of all-cause mortality (4). Currently, continuous positive airway pressure (CPAP) remains the gold standard of treatment; however, its clinical efficacy is often severely limited by suboptimal patient adherence (5). More importantly, a recent meta-analysis by Fletcher et al. (6) indicated that while CPAP therapy effectively improves airway patency, it is insufficient to significantly enhance a patient's maximal oxygen consumption (VO^2^max) or anaerobic threshold (AT) (6). These findings suggest that mechanical ventilation alone cannot fully restore the impaired physiological exercise capacity of patients, necessitating the integration of more active “exercise training.”

In light of the limitations of CPAP, clinical research has begun exploring non-mechanical alternative therapies. For instance, a systematic review published by Rueda et al. (7) confirmed that myofunctional therapy targeting the oropharynx is effective in reducing the apnea-hypopnea index (AHI) and improving sleepiness (8). Nevertheless, such interventions primarily focus on localized muscle function training and fail to address the core issues of sedentary behavior and generalized low cardiorespiratory fitness prevalent among OSA patients (9). As emphasized by Fletcher et al., achieving substantial improvements in oxidative capacity and physical fitness requires breaking the sedentary cycle. Consequently, there is an urgent clinical need for comprehensive intervention strategies capable of effectively promoting systemic physical activity and enhancing metabolic health.

The exponential growth of digital health technologies (DHTs) offers an innovative pathway to fill this management gap. These technologies encompass a wide range of tools, from telemedicine platforms and wearable devices to mobile health (mHealth) applications, extending far beyond the scope of traditional monitoring (10, 11). As noted in the study by Kim et al. (13), the market for such devices including smart wearables and mobile apps is growing exponentially, and their integrated sensors (e.g., oximeters, accelerometers) are demonstrating increasing accuracy in large-scale screening for sleep disorders (12, 13). Crucially, unlike traditional passive monitoring, these digital technologies possess the unique capability to provide real-time behavioral feedback and gamified incentives, which can theoretically be transformed into active intervention tools for promoting lifestyle changes (14). The review by Lugo et al. (15) also highlighted the potential of telemedicine to enhance adherence in chronic disease management, although its application in the domain of physical activity remains under-explored (15).

Despite the increasing maturity of these technologies, evidence regarding the efficacy of digital interventions remains inconclusive. Previous studies have largely relied on subjective questionnaires (e.g., IPAQ) to assess physical activity, which are prone to significant recall bias and fail to reflect actual changes in exercise volume. Furthermore, while Pires et al. focused on the diagnostic accuracy of these technologies, high-quality evidence remains lacking regarding their role as “intervention tools” in driving objective physical activity indicators (e.g., step counts) and improving cardiorespiratory fitness (VO^2^max) (16). Based on this, the present study aims to evaluate the impact of digital health technology-based interventions on objective physical activity levels, cardiorespiratory fitness, and sleep-respiratory parameters in OSA patients through a systematic review and meta-analysis. Additionally, this study will deeply explore the differences across various intervention modes regarding theoretical drivers, feedback mechanisms, and the application of behavior change techniques (BCTs) to identify the core elements that support behavioral changes in this population.

## 2 Methods

This systematic review and meta-analysis was written and reported in strict accordance with the Preferred Reporting Items for Systematic Reviews and Meta-Analyses (PRISMA 2020) statement. The study protocol was prospectively registered on the International Prospective Register of Systematic Reviews (PROSPERO) [Registration number: (CRD420251246601)] to establish the transparency and standardization of the study (17).

### 2.1 Search strategy

This study conducted a comprehensive search on November 19, 2025, across PubMed, Embase, Web of Science, The Cochrane Library, and CINAHL electronic databases. The search coverage extended from the inception of each database to the date of the search. Additionally, to minimize publication bias, this study performed an extra search on the ClinicalTrials.gov registration platform to identify ongoing or unpublished clinical trials. The search strategy strictly followed the PICOS principle, using a combination of Medical Subject Headings (MeSH) and free-text terms. Boolean operators “AND” were used to concatenate three core concepts: terms covering the target population of obstructive sleep apnea, terms involving digital health and telemedicine intervention technologies, and terms related to physical activity and lifestyle intervention content. By setting study type filters, this study explicitly limited inclusion to randomized controlled trials, strictly excluding observational studies, reviews, and other non-experimental designs. Search syntax was adjusted according to the characteristics of different databases; detailed search strategies and specific search formulas for each database are provided in Annex 1.

### 2.2 Inclusion and exclusion criteria

The study population consisted of adults aged 18 years and older with a confirmed diagnosis of obstructive sleep apnea. To reduce confounding factors, patients with comorbidities limiting limb movement, such as recent stroke, heart failure, or severe neuromuscular diseases, were excluded. Regarding interventions, the study selected those based on digital health technologies, specifically including smartphone applications, wearable devices, telemedicine platforms, or web-based programs. Included intervention protocols were required to contain components targeting physical activity, exercise training, or lifestyle management, implemented either via active guidance or passive monitoring and feedback. Studies focusing solely on remote monitoring of CPAP adherence without any physical activity monitoring or intervention components were excluded. The comparator groups were defined as those receiving standard care, wait-list control, or sham intervention without feedback. For outcomes, studies were required to include at least one primary outcome, namely objective physical activity indicators such as daily steps, VO2max, or 6-min walk distance, or secondary outcomes including body mass index, body weight, apnea-hypopnea index, and Epworth Sleepiness Scale scores. Finally, regarding study design, only randomized controlled trials published in English were included (18).

### 2.3 Data extraction and quality assessment

Initial screening and full-text review of literature were performed independently by two researchers, with any discrepancies resolved through discussion or consultation with a third-party arbitrator. Data extraction utilized standardized Excel forms to strictly record basic study characteristics (including first author and publication year), baseline participant characteristics (covering sample size and disease severity), specific digital intervention modes, and mean and standard deviation (SD) of outcome data at various time points. The risk of bias for included studies was assessed using the Cochrane Risk of Bias tool (RoB 2.0) (Cochrane, London, UK), evaluating five domains: randomization process, deviations from intended interventions, missing outcome data, measurement of the outcome, and selection of the reported result. Furthermore, this study employed the Grading of Recommendations Assessment, Development and Evaluation (GRADE) system to grade the certainty of evidence for outcome indicators such as daily steps and AHI, comprehensively considering risk of bias, inconsistency, indirectness, imprecision, and publication bias (19).

### 2.4 Statistical analysis

All statistical analyses were performed using Review Manager software version 5.4 from The Cochrane Collaboration. For continuous variables, effect sizes were expressed as mean differences with 95 percent confidence intervals if the same measurement tools were used across studies, such as for body mass index, step count, and the apnea-hypopnea index. Otherwise, standardized mean differences were employed. For studies that did not directly report the standard deviation of the change scores, the standard deviations were estimated based on baseline and endpoint data by assuming a pre-post correlation coefficient of 0.5 in accordance with the recommendations of the Cochrane Handbook for Systematic Reviews of Interventions. Inter-study heterogeneity was assessed using the chi-square test and the I^2^ statistic. This study systematically reports the I^2^ value for each outcome to quantify the impact of heterogeneity on the pooled results. Given the potential methodological variations across studies, including differences in physical activity measurement devices such as research-grade accelerometers and commercial smartbands, as well as variations in data collection protocols and outcome definitions, all meta-analyses were uniformly conducted using the more conservative random-effects model to obtain more generalizable effect size estimates and to compensate for potential heterogeneity (20). To ensure transparency and reproducibility, the subgroup analysis protocol was pre-specified prior to data analysis. The classification of intervention modes into active intervention and passive monitoring was based on theoretical frameworks of digital health interventions concerning interaction depth and feedback mechanisms (21). Active intervention was defined as a mode involving bidirectional interaction, delivery of behavior change techniques, and real-time feedback. In contrast, passive monitoring focused on unidirectional data tracking without real-time interactive reminders (22). To minimize analyst subjectivity, two researchers independently categorized the included studies according to these pre-specified criteria, and any discrepancies were resolved through consensus-based discussion with a third senior researcher. Subgroup analyses based on intervention modes were pre-specified to explore potential sources of heterogeneity. Additionally, considering the influence of intervention duration on the consolidation of behavior change, an exploratory subgroup analysis was conducted using a 3-month cutoff to examine the potential impact of intervention duration. Statistical significance was set at a two-sided *P* < 0.05. Since the number of studies included in the meta-analysis for each outcome was less than 10, funnel plot analysis for evaluating publication bias was not performed (23).

## 3 Results

### 3.1 Literature screening process

**Figure 1:**
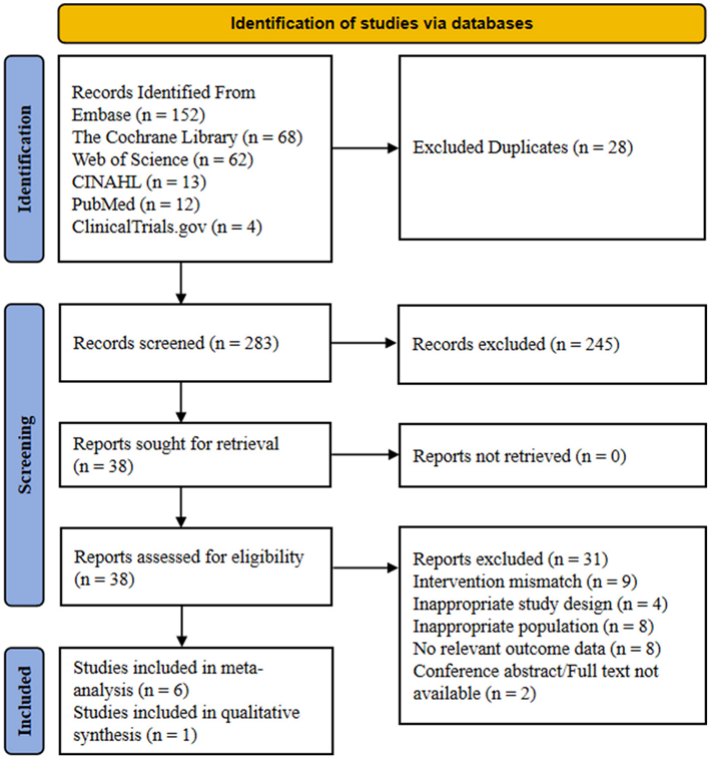
Preferred reporting items for systematic reviews and meta-analysis (PRISMA) study flow diagram.

The initial search yielded a total of 311 records, comprising 152 from Embase (Elsevier, Amsterdam, The Netherlands), 68 from the Cochrane Central Register of Controlled Trials, 62 from Web of Science, 13 from CINAHL, 12 from PubMed, and four from ClinicalTrials.gov. After removing 28 duplicates using EndNote (Clarivate, Philadelphia, USA) 21 software followed by manual verification, 283 records remained for title and abstract screening. Following independent screening by two reviewers, 245 records clearly not meeting inclusion criteria were excluded, and the full texts of the remaining 38 articles were retrieved for detailed assessment. During the full-text assessment phase, 31 articles were excluded for the following reasons: nine studies presented an intervention mismatch such as monitoring only CPAP adherence without physical activity components, four studies had an inappropriate study design including non-RCTs or single-arm studies, eight studies involved an inappropriate population primarily involving patients with severe stroke or heart failure, eight studies were missing outcome data lacking key data such as steps, BMI, or AHI, and two studies were excluded due to publication type mismatch as they were conference abstracts. Ultimately, seven randomized controlled trials were included in the systematic review, of which six were included in the quantitative meta-analysis. The literature screening process is detailed in Figure 1.

### 3.2 Characteristics of included studies

A total of seven randomized controlled trials (RCTs) published between 2014 and 2024 were finally included in this study (24–30). The total sample size across the included studies was 693 patients with confirmed obstructive sleep apnea (OSA), with individual study sample sizes ranging from 28 to 306 participants (25, 26). The participants were primarily middle-aged, with mean ages ranging from 38.3 to 62.0 years (29, 30), and generally presented with overweight or obese characteristics, as indicated by baseline mean body mass index (BMI) values between 27.9 and 32.4 kg/m^2^ (25, 27). Baseline disease severity ranged from mild-to-moderate to severe OSA, with the mean apnea-hypopnea index (AHI) fluctuating between 17.2 and 46.0 events/hour (25, 30).

Regarding intervention characteristics, the included studies utilized diverse digital health technologies. Based on theoretical foundations and behavior change techniques (BCTs), this study further refined the intervention measures into four categories (31). The first category is the Theory-driven and Education-enhanced mode, which involved two studies (27, 30); these intervention protocols were strictly based on behavior change theories (such as self-management models) and deeply integrated multiple BCTs, including goal setting, multi-dimensional self-monitoring, and social support. The second category is the Clinically-integrated and Synchronous Interactive mode, also involving two studies (24, 28), characterized by deep interaction through shared data between clinicians and patients (e.g., linkage with hospital EMR) or real-time professional demonstration and supervision via synchronous live-streamed sessions. The third category is the Basic digital feedback and Monitoring-focused mode, involving two studies (25, 26), which primarily focused on automated clinical feedback generated by devices (e.g., air leaks, blood pressure) or simple recording based on lifelogging, though lacking a clear behavioral coaching logic. Finally, the Passive Monitoring mode involved only one study by Mendelson et al. (29), where the intervention was limited to unidirectional remote transmission of physiological data without providing feedback or intervention for behavioral indicators such as physical activity or weight management. Intervention durations ranged from 4 weeks to 6 months (27, 30). Control groups primarily received standard care or routine health education, with one study employing an active control design involving the wearing of devices (27). The primary outcomes reported across the included studies encompass objective physical activity levels primarily measured by daily step counts, cardiorespiratory fitness comprising peak oxygen uptake and the 6-min walk distance, body composition indices including body mass index and body weight, and clinical severity markers such as the apnea-hypopnea index and blood pressure. During the data extraction process for continuous variables, for studies that did not directly report the standard deviation of change, estimations were performed based on baseline and endpoint data. Among the seven trials included in this study, the correlation coefficient imputation method was employed to obtain the necessary statistical data. The detailed characteristics are summarized in Table 1.

**Table 1:** Characteristics of the included studies.

**Study ID**	**Sample size (I/C)**	**Population baseline (mean ±SD)**	**Refined intervention category**	**Theoretical basis**	**Key functional components and BCTs**	**Duration**	**Key outcomes**
Petrov et al. (24)	40/47	Age: 57.6 ± 12.3 AHI: 25.2 ± 21.8 BMI: 32.4 ± 6.1	Theory-driven active	Stepnowsky's self-management model	SleepWell24 app: self-monitoring (PAP/PA/Sleep), Personalized feedback, Goal-setting, Troubleshooting toolbox	60 days	PAP Adherence, BMI
Eyuboglu et al. (28)	22/22	Age: 43.5 ± 8.0 AHI: 28.3 ± 19.5 BMI: 31.8 ± 4.7	Synchronous interactive active	Not explicitly stated (yoga principles)	Tele-yoga via Zoom: real-time demonstration, Professional instruction, Group-based social support, Synchronous feedback	12 weeks	VO2 max, 6MWT, ESS
Cho et al. (27)	24/23	Age: 43.0 ± 9.5 AHI: 21.9 ± 15.8 BMI: 27.9 ± 3.1	Clinically-integrated active	Not explicitly stated (EMR-linked model)	Smartphone app + Misfit tracker: integrated with hospital EMR, Physician-led data review, Personalized goal-setting	4 weeks	BMI, AHI, Snoring
Lin et al. (30)^*^	34/40	Age: 38.3 ± 6.2 AHI: 17.2 ± 11.4 BMI: 28.6 ± 3.6	Education-enhanced active	Adjusting lifestyle intervention (ALI)	MyFitnessPal log + Health education lectures, Social support (online community), Weekly counselor feedback	6 months	BMI, AHI, WC
Kim et al. (26)^*^	15/13	Age: 43.1 ± 8.7 AHI: NR BMI: 28.9 ± 2.8	Engagement-driven active	Patient activation and lifelogging	MyHealthKeeper app: manual log (diet/stress/sleep), Step feedback, Clinician comments for behavior reinforcement	4 weeks	Steps, Weight
Pépin et al. (25)	157/149	Age: 60.8 ± 10.0 AHI: 46.0 ± 19.0 BMI: 32.4 ± 5.0	Monitoring-focused active	Not explicitly stated	Multimodal telemonitoring: automatic feedback on leaks/BP/activity, Side-effect management algorithms, No behavioral coaching	6 months	Steps, BMI, BP, ESS
Mendelson et al. (29)	54/53	Age: 62.0 ± 9.0 AHI: 39.0 ± 16.7 BMI: 29.9 ± 4.8	Passive monitoring	Not explicitly stated	Telemedicine platform: passive data transmission (BP/CPAP), Daily pictograms for symptoms, No feedback on physical activity	4 months	Steps, BMI, BP, ESS

Sample size refers to the number of participants analyzed. ^*^For Kim et al. and Lin et al., sample sizes represent the specific intervention arms selected for meta-analysis (App + Wearable and App + Education, respectively). RCT, randomized Controlled Trial; I/C, intervention/control; BMI, body mass index; AHI, apnea-hypopnea index; WC, waist circumference; BP, blood pressure; ESS, epworth sleepiness scale; 6mwt, 6-min walk test; Vo2max, maximal oxygen consumption; NR, not reported.

### 3.3 Risk of bias assessment

**Figure 2:**
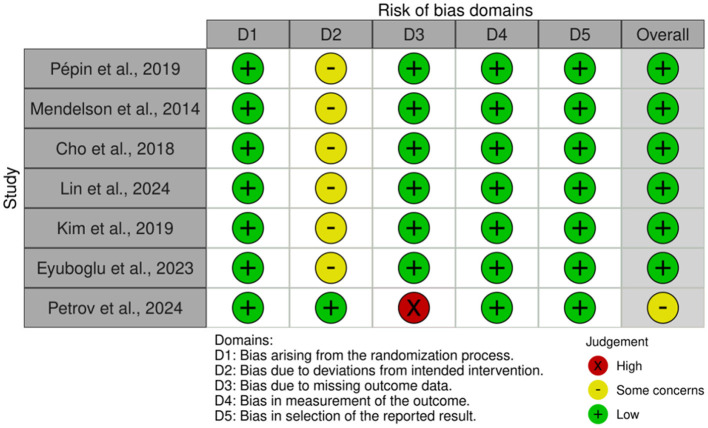
Risk of bias summary: review of the authors judgments about each risk of bias item for each included study.

**Figure 3:**
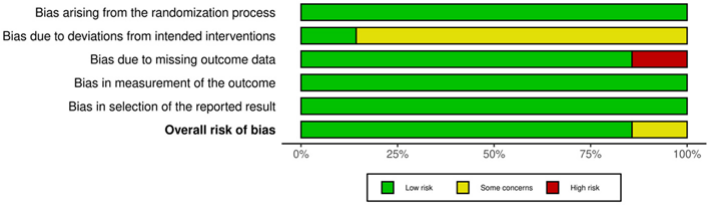
Risk of bias graph: review authors' judgments about each risk of bias item, presented as percentage of included studies.

The risk of bias assessment results for the included studies are detailed in Figures 2, 3. This study used the Cochrane RoB 2.0 tool to evaluate the 7 RCTs. Overall, the methodological quality of the included literature was high, with no systemic biases identified that would seriously undermine the validity of the conclusions. Specifically, all seven studies were rated as low risk in the three critical domains of randomization process, measurement of the outcome, and selection of the reported result, indicating high rigor in random sequence generation, allocation concealment, objective data collection, and execution of pre-registered protocols, providing a robust evidence base for the meta-analysis. Despite the high overall quality, specific limitations existed in certain domains. Regarding deviations from intended interventions, due to the inherent nature of digital health and behavioral interventions, it was difficult to blind participants. Consequently, except for one study employing an active control design (24), the remaining six studies were rated as having some risk. However, given that this study primarily focused on objective physiological indicators including BMI and AHI, the interference of such implementation bias on results is relatively limited. Regarding missing outcome data, the vast majority of studies effectively controlled for attrition bias through intention-to-treat analysis, with only one study marked as high risk due to a high rate of data loss. In summary, despite objective limitations in blinding implementation, the overall quality of evidence for the included studies remains reliable.

### 3.4 Meta-analysis results

#### 3.4.1 Objective physical activity and cardiorespiratory fitness

**Figure 4:**
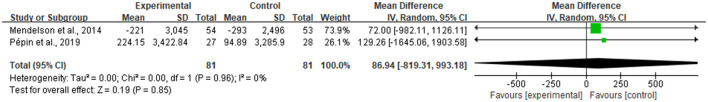
Forest plot of daily steps.

Regarding daily step counts, the quantitative synthesis included two randomized controlled trials representing a total of 162 participants. The pooled analysis, as illustrated in Figure 4, demonstrated that the difference in the change of daily step counts between the intervention and control groups was 86.94 steps with a 95 percent confidence interval ranging from −819.31 to 993.18 and a *P*-value of 0.85. The inter-study heterogeneity was observed to be 0 percent. Although the overall pooled results did not show a significant difference, the study by Kim et al. in 2019 specifically reported absolute step counts during the intervention period, showing that the daily average steps in the group using a combination of applications and wearable devices reached 8,165 steps, which was significantly higher than the 6,034 steps observed in the group using applications alone with a *P*-value of 0.02. This finding indicates potential additional benefits that may be derived from device-assisted active monitoring.

Regarding cardiorespiratory fitness and functional exercise capacity, data from the study by Eyuboglu et al. (28) showed that the improvement in VO^2^max in the intervention group was 342.41 ml/min higher than in the control group [95% CI (38.05, 646.77)], a difference that was statistically significant (28). However, in terms of functional exercise capacity measured by 6-min walk distance, although the intervention group showed an improvement trend (MD = 20.37 m), this difference did not reach statistical significance [95% CI (−21.49, 62.23)].

#### 3.4.2 Body composition

**Figure 5:**
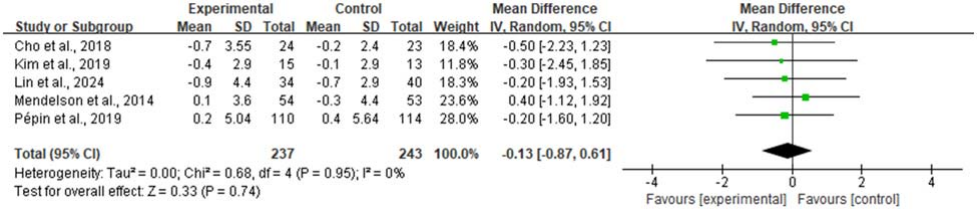
Forest plot of body mass index (BMI).

For the improvement of Body Mass Index (BMI), the meta-analysis included five RCTs (Figure 5), totaling 480 participants (237 in intervention, 243 in control). The pooled analysis results indicated that although the magnitude of BMI reduction in the intervention group was slightly larger than in the control group, the between-group difference did not reach statistical significance [MD = −0.13 kg/m^2^, 95% CI (−0.87, 0.61), *P* = 0.74]. Notably, there was good homogeneity among studies, with heterogeneity test results showing an I^2^ of 0%.

**Figure 6:**
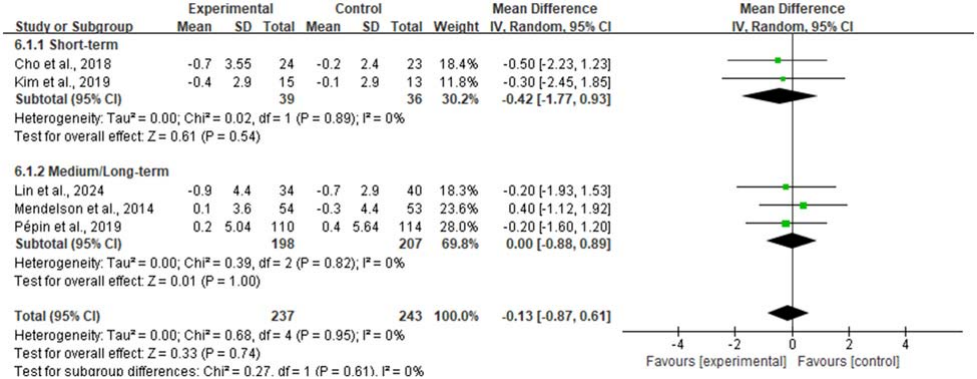
Exploratory subgroup analysis based on intervention duration.

To explore whether intervention duration affects weight loss outcomes, this study conducted an exploratory subgroup analysis based on duration, divided into short-term (< 3 months) and medium-to-long-term (≥3 months) groups. As shown in Figure 6, the short-term intervention group exhibited a larger trend of BMI reduction (MD = −0.42 kg/m^2^), while the medium-to-long-term group showed no overall change. However, the test for subgroup differences did not reach statistical significance, indicating that intervention duration may not be the primary factor determining differences in BMI improvement.

**Figure 7:**
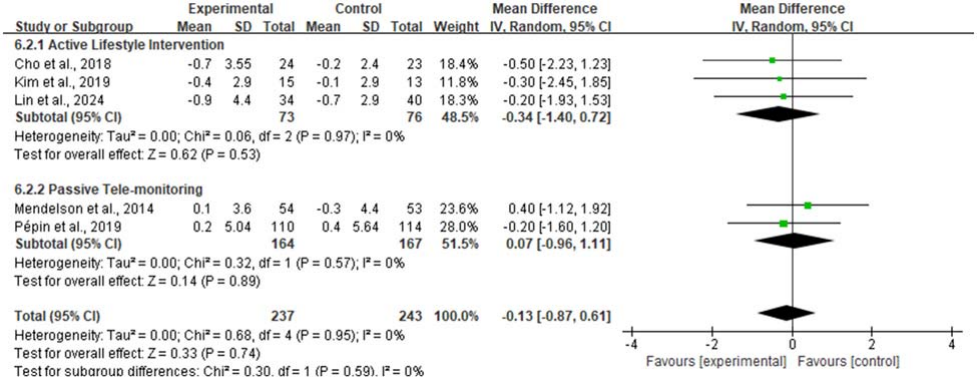
Subgroup analysis based on intervention type.

Further subgroup analysis categorized interventions based on interaction mode into Active Lifestyle Intervention and Passive Tele-monitoring. Results shown in Figure 7 indicated that the active intervention subgroup, involving self-monitoring and feedback, numerically showed a tendency for BMI reduction (MD = −0.34 kg/m^2^), while the passive monitoring subgroup, involving only data transmission, showed a slight numerical increase (MD = 0.07 kg/m^2^). Although the statistical test for subgroup differences did not reach significance (*P* = 0.59), indicating that a fundamental difference in efficacy between the two modes cannot currently be concluded, the directional difference in effect sizes between the two groups suggests that intervention modes emphasizing active engagement and behavioral change may have potential clinical value. This hypothesis merits further verification in larger sample studies. Additionally, heterogeneity within both subgroups was 0%, indicating high consistency of effects within each intervention mode type.

**Figure 8:**
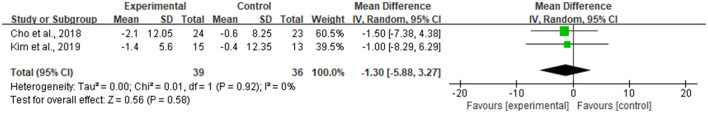
Forest plot of body weight.

Furthermore, analysis regarding absolute Body Weight included two studies (see Figure 8). Results similarly showed a weight loss trend favoring the intervention group [Mean Difference = −1.30 kg, 95% CI (−5.88, 3.27)], but the difference did not reach statistical significance (*Z* = 0.56, *P* = 0.58).

#### 3.4.3 Clinical symptoms and disease severity

**Figure 9:**
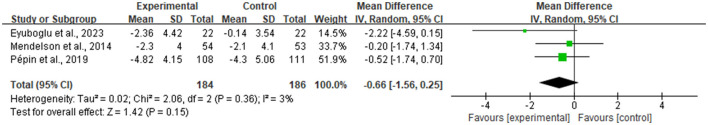
Forest plot of the epworth sleepiness scale (ESS).

**Figure 10:**
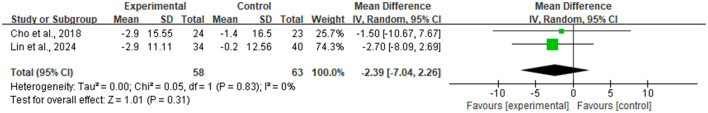
Forest plot of the apnea-hypopnea index (AHI).

Regarding the improvement of daytime sleepiness symptoms, three trials were included (Figure 9), involving 370 participants. Although the reduction in Epworth Sleepiness Scale (ESS) scores in the intervention group was numerically superior to the control group, pooled analysis indicated that the between-group difference did not reach statistical significance [MD = −0.66, 95% CI (−1.56, 0.25), *P* = 0.15], with extremely low heterogeneity between studies (I^2^ = 3%). Regarding the improvement of the Apnea-Hypopnea Index (AHI), two trials were included (Figure 10), involving 121 participants. Pooled results showed a decreasing trend in the index for the digital health intervention group (MD = −2.39 events/h), but the difference did not reach statistical significance [95% CI (−7.04, 2.26), *P* = 0.31].

In terms of cardiovascular risk indicators, data based on intention-to-treat analysis by Pépin et al. (25) showed that the difference in change of morning systolic blood pressure between the intervention and control groups was 0.71 mmHg [95% CI (−3.46, 4.88)], with no statistical difference observed (25). Mendelson et al. (29) similarly found no statistical difference between groups in blood pressure changes reported in subgroup analyses and charts (29).

### 3.5 Quality of evidence

This study used the GRADE system to assess the certainty of evidence for five key outcome indicators; detailed grading results are shown in Table 2. Overall, the quality of evidence ranged from “Moderate” to “Low.” Evidence quality for BMI, ESS, and AHI was rated as “Moderate,” while evidence for daily steps and body weight was rated as “Low.” The primary factor leading to evidence downgrading was Imprecision, i.e., the 95% confidence intervals of the pooled effect sizes were wide and included the null value. Additionally, daily steps were further downgraded for risk of bias due to high data missing rates in included studies; body weight was judged to have serious imprecision due to a very small total sample size (< 200) and extremely wide confidence intervals. Given that the number of studies included in the meta-analysis for each outcome indicator was fewer than 10, publication bias was not assessed. Despite these limitations, the extremely low heterogeneity among studies indicates a high consistency in the direction of intervention effects.

**Table 2:** GRADE evidence quality evaluation.

**Outcomes**	**No. of studies (participants)**	**Risk of bias**	**Inconsistency**	**Indirectness**	**Imprecision**	**Publication bias**	**Quality of evidence**
Daily steps	2 (162)	Downgraded^a^	Not downgraded^b^	Not downgraded	Downgraded^c^	Not assessed e	⊕⊕○○ Low
BMI	5 (480)	Not downgraded	Not downgraded^b^	Not downgraded	Downgraded^c^	Not assessed^e^	⊕⊕⊕○ Moderate
Body weight	2 (75)	Not downgraded	Not downgraded^b^	Not downgraded	Seriously downgraded^d^	Not assessed^e^	⊕⊕○○ Low
ESS	3 (370)	Not downgraded	Not downgraded^b^	Not downgraded	Downgraded^c^	Not assessed^e^	⊕⊕⊕○ Moderate
AHI	2 (121)	Not downgraded	Not downgraded^b^	Not downgraded	Downgraded^c^	Not assessed^e^	⊕⊕⊕○ Moderate

a: downgraded for risk of bias due to high rates of missing data regarding step counts in the included studies, suggesting potential attrition bias. b: I^2^ >50% (Note: no downgrade for inconsistency was applied as I^2^ values were consistently low across all outcomes in this study). c: downgraded for imprecision as the 95% confidence interval crosses the line of no effect (includes 0). d: downgraded for serious imprecision due to small total sample size (< 200 participants across only 2 studies) and wide confidence intervals. e: publication bias was not assessed via funnel plots or Egger's test because fewer than 10 studies were included.

## 4 Discussion

### 4.1 Findings

This systematic review and meta-analysis evaluated the comprehensive intervention effects of digital health technologies on patients with obstructive sleep apnea based on pooled data from 7 randomized controlled trials. In terms of overall effects, compared with standard care, digital health interventions did not show statistically significant differences in improving objective daily steps, reducing BMI, improving AHI, or alleviating daytime sleepiness. Based on the GRADE system assessment, the certainty of evidence for the aforementioned main outcomes generally ranged from moderate to low levels; the primary factors for downgrading were imprecision of effect estimates and risk of missing data in some studies. Although overall results did not reach statistical significance, this study captured signals of potential physiological benefits in exploratory analyses. Included studies showed that tele-rehabilitation-based active intervention significantly improved VO^2^max; meanwhile, subgroup analysis results numerically suggested that active intervention modes integrating real-time feedback might be superior to simple passive monitoring in improving BMI and steps. Given the limited quantity of current evidence and complex sources of heterogeneity, these observational findings regarding the advantages of active intervention are still in a preliminary stage and require verification by more high-quality randomized controlled trials.

### 4.2 Comparison with previous studies and mechanism analysis

The overall statistically non-significant results of this study diverge from the findings of some earlier observational studies. Through an in-depth comparison with existing literature, we identified those discrepancies in interaction depth and the theoretical basis of intervention models are the primary drivers of this divergence. The lack of significant differences in step counts and body mass index (BMI) in this meta-analysis is largely attributable to several heavily weighted studies that primarily employed passive remote monitoring modes (29). While previous reviews by Lugo et al. affirmed the role of telemedicine in enhancing adherence to continuous positive airway pressure (CPAP) management (15), our findings further suggest that this passive focus on unidirectional physiological data transmission appears insufficient to drive patients to break deep-rooted sedentary lifestyle cycles (31). This limitation reflects functional heterogeneity within “active” interventions: for instance, Pépin et al. (25, 29), which carries substantial weight in our analysis, possesses automated feedback features; however, its “monitoring-oriented” nature lacks the behavioral change logic necessary to effectively improve physical activity. Conversely, the study by Petrov et al. (24, 30) included in our review found that although objective physical activity and self-efficacy showed no significant short-term differences, patients in the intervention group demonstrated significant improvements in perceived risk. This suggests that such “theory-driven” models, by integrating core behavior change techniques (BCTs) such as goal setting and troubleshooting, may initiate cognitive remodeling prior to observable changes in objective behavior (32). In stark contrast, existing randomized controlled trials have directly validated the value of interactive technologies (33, 34); when wearable devices are integrated with applications to provide real-time feedback, patients' mean daily step counts were significantly higher than those in passive recording groups using apps alone. Furthermore, our subgroup analysis observed a trend toward greater BMI reduction in the active intervention group compared to the passive monitoring group. These contrasting results suggest that the controversy surrounding the efficacy of digital interventions in the literature may stem from a conflation of “passive monitoring” and “active intervention” across different studies. To achieve substantial therapeutic effects, intervention tools must evolve from simple recording functions toward active systems that provide goal setting and real-time feedback (35, 36).

Although this study did not observe significant improvements in AHI, this does not diminish the unique clinical value of digital interventions, particularly their role in filling gaps in physiological adaptation left by standard treatments. Mendelson et al. (29) pointed out that while CPAP therapy effectively addresses airway collapse, its impact on enhancing cardiorespiratory fitness indicators, such as maximal oxygen consumption, is quite limited. It must be combined with exercise training to effectively restore impaired cardiorespiratory fitness in patients; the improvement in maximal oxygen consumption (VO2max) observed in this study directly addresses this clinical need (37). This significant physiological benefit can be attributed to the inclusion of high-intensity interactive components. For example, the “synchronous interactive mode” utilized by Eyuboglu et al. (28) provided real-time professional demonstration, guidance, and social support via remote live-streaming, enabling a higher level of behavioral rehearsal within the BCT framework and resulting in significant VO^2^max gains. Simultaneously, Carneiro-Barrera et al. (38) emphasized that standard treatments focusing solely on localized airway obstruction often fail to resolve the systemic metabolic regulation impairments common in OSA patients (38). Therefore, systemic aerobic exercise guided by digital health effectively addresses the systemic cardiorespiratory fitness deficit remaining after CPAP resolves airway patency and myofunctional training addresses localized muscle tension. For the high-risk OSA population, improving cardiorespiratory fitness is an independent predictor of reduced all-cause mortality, and its long-term cardiovascular protective significance may outweigh short-term changes in AHI (39, 40).

From a methodological perspective, the inter-study statistical heterogeneity for the vast majority of outcome measures, including daily step counts, body mass index, and the apnea-hypopnea index, remained at an extremely low level, which was predominantly zero percent. Despite inherent differences among the included studies regarding objective measurement devices, such as research-grade accelerometers and commercial smartbands, as well as variations in data collection protocols, the high degree of statistical homogeneity suggests that the improvement effects of digital health interventions for patients with obstructive sleep apnea possess favorable stability across various technical platforms. However, considering the relatively small number of studies currently included, the statistical power of heterogeneity tests may be limited, making it difficult to entirely rule out potential clinical heterogeneity arising from differences in device precision. Consequently, this study maintained the use of the random-effects model for conservative estimation and incorporated detailed subgroup analyses to ensure that the interpretation of pooled effect sizes remains robust and reliable within the context of potential methodological discrepancies.

### 4.3 Research limitations

This study possesses several limitations primarily stemming from the characteristics of the existing primary evidence and the inherent challenges within the digital health field. First, to ensure the objectivity of the evidence, this study strictly restricted inclusion to trial data based on objective measurement tools such as accelerometers, which resulted in a relatively limited number of randomized controlled trials meeting the inclusion criteria. The insufficient sample size not only constrained the statistical power for pooling certain outcome measures but also increased the risk of Type II errors in subgroup analyses, namely the possibility that true differences between active intervention and passive monitoring were not detected due to inadequate statistical power. Simultaneously, the limited number of included studies precluded an effective assessment of publication bias via funnel plots, suggesting that unpublished negative results might have a potential influence on the pooled effect sizes.

Second, a certain degree of uncertainty exists regarding the statistical processing of this study. Given that some primary studies did not report the standard deviation of change scores before and after the intervention, data imputation was performed by assuming a correlation coefficient of 0.5 according to the methods recommended in the Cochrane Handbook. Although this is a widely recognized statistical compensatory technique, such hypothetical estimation may, to some extent, influence the precision of the confidence intervals for the pooled effect sizes. Future research should more formally report original change values and their degree of variation to further enhance the statistical rigor of the conclusions.

Furthermore, this study primarily focused on objectively measured physiological and behavioral outcomes and did not perform a quantitative synthesis of psychological mediating variables in the process of behavior change, such as self-efficacy and health beliefs. Behavioral evidence suggests that the remodeling of patient cognition is often a precursor to substantial shifts in objective behavior (41). Furthermore, this study only included literature published in English. Although previous methodological research indicates that language restrictions have limited impact on meta-analyses of conventional medical interventions, the possibility of language bias cannot be entirely excluded. Third, there is inherent heterogeneity among the included studies regarding digital intervention technologies. Despite subgroup analyses being conducted to distinguish between intervention modes, differences in specific behavior change techniques, interaction frequencies, and device sensor precision across studies may still introduce potential methodological influences. Moreover, due to the nature of behavioral interventions, it is difficult to implement double-blinding for participants and implementers. Although this study focused on objective physiological indicators to reduce measurement bias, the potential risk of implementation bias cannot be completely ruled out. Finally, the follow-up periods in the included studies were primarily concentrated in the short-to-medium term, lacking definitive evidence regarding the maintenance of long-term lifestyle changes and long-term cardiovascular benefits. Future research needs to extend follow-up durations to evaluate the sustained efficacy of digital therapies.

### 4.4 Practical implications

The findings of this study provide a preliminary perspective on the comprehensive management of chronic obstructive sleep apnea, suggesting that future clinical strategies may need to extend from simple remote monitoring to more interactive active intervention models. First, when interpreting the efficacy results of this study, the association between statistical significance and clinically relevant differences warrants further exploration. Although this meta-analysis shows that digital health technology has not yet reached statistical significance in improving daily step counts and BMI, the magnitude of these changes appears not to have reached the established thresholds for Minimal Clinically Important Difference (MCID), such as an increase of 1,000 steps per day or a BMI reduction of 1 kg/m^2^ (42, 43). This tentatively suggests that current digital protocols may still have limitations in driving “clinically effective” lifestyle changes; future intervention designs should perhaps aim to reach the MCID by attempting to intensify the application of behavior change techniques (BCTs) to observe whether more clinically meaningful behavioral changes can be produced.

Second, clinical intervention design should consider shifting from passive monitoring toward theory-driven active interaction models. Given that current evidence tends to show potential limitations of passive data recording in driving behavior change, clinical protocols should try to prioritize tools that integrate wearable sensors and possess closed-loop feedback mechanisms. Particularly for patients with poor CPAP adherence or milder symptoms, such digital health-based interventions could serve as a potential adjunctive measure. Additionally, because changes in objective behavior may often lag behind cognitive reconstruction, clinical assessment should consider observing changes in psychological mediating indicators, such as a patient's perceived risk of the disease, alongside objective metrics, as this might provide clues for evaluating early intervention effects (44).

Finally, digital health interventions demonstrate potential value in filling the physiological benefit gaps of traditional treatments. Although the improvement of digital interventions on AHI has not yet been confirmed, the positive signal shown in maximal oxygen consumption suggests its potential significance in improving cardiorespiratory fitness, which may offer a supplementary approach for cardiovascular risk management in OSA patients. However, it must be noted that due to the limitations in the number of included studies and follow-up periods, the results of this study should be interpreted with caution. The actual efficacy of digital health technology in filling the gaps of traditional treatments still requires further verification in future larger-scale, rigorously designed multi-disciplinary collaborations that include exercise physiology guidance.

## 5 Conclusion

This systematic review indicates that current evidence is insufficient to support the notion that digital health technology can significantly improve objective physical activity levels, body composition, or disease severity in patients with obstructive sleep apnea. This may be largely attributed to the prevalence of passive monitoring modes that lack deep interaction in existing studies. Nevertheless, the physiological benefits shown in the improvement of maximal oxygen consumption reveal its unique value in enhancing cardiorespiratory fitness. Simultaneously, subgroup analysis results tentatively suggest that intervention effects are closely related to their theoretical basis and functional depth; active models characterized by synchronous interaction (e.g., real-time remote guidance) or theory-driven components (e.g., integrated BCTs like goal setting and self-monitoring) demonstrate significant potential in driving behavior change compared to simple passive monitoring. In view of this, future clinical practice and research should consider shifting toward active interactive digital therapies that integrate core behavior change techniques (BCTs) and possess clear theoretical drivers, while exploring their integration as a key adjunctive strategy within multi-disciplinary treatment systems to provide a new pathway for further improving the long-term cardiovascular prognosis of patients beyond traditional treatments.
