# Supplementary material for: From passive monitoring to active engagement: a systematic review and meta-analysis of digital health technologies for improving objective physical activity and cardiorespiratory fitness in patients with obstructive sleep apnea
Source: Front Public Health. 2026 Feb 27;14:1760571. doi: 10.3389/fpubh.2026.1760571 (PMC12982425; doi:10.3389/fpubh.2026.1760571)
Supplement: Supplementary file 1 [file Supplementary_file_1.docx]

**Supplementary Text 1 Search Strategies**

1. **PubMed：**

search strategy：

#1 ("Sleep Apnea, Obstructive"[Mesh] OR "Obstructive Sleep Apnea"[Title/Abstract] OR "OSA"[Title/Abstract] OR "Sleep Apnoea"[Title/Abstract] OR "Sleep Disordered Breathing"[Title/Abstract] OR "Sleep Hypopnea"[Title/Abstract])

#2 ("Telemedicine"[Mesh] OR "Mobile Applications"[Mesh] OR "Wearable Electronic Devices"[Mesh] OR "Internet-Based Intervention"[Mesh] OR "Digital health"[Title/Abstract] OR "mHealth"[Title/Abstract] OR "eHealth"[Title/Abstract] OR "Telehealth"[Title/Abstract] OR "Telemedicine"[Title/Abstract] OR "Smartphone"[Title/Abstract] OR "Mobile app*"[Title/Abstract] OR "Wearable*"[Title/Abstract] OR "Activity tracker"[Title/Abstract] OR "Pedometer"[Title/Abstract] OR "Remote monitoring"[Title/Abstract] OR "Web-based"[Title/Abstract] OR "Online program"[Title/Abstract] OR "Virtual"[Title/Abstract])

#3 ("Exercise"[Mesh] OR "Sports"[Mesh] OR "Physical Exertion"[Mesh] OR "Physical functional performance"[Mesh] OR "Life Style"[Mesh] OR "Diet, Reducing"[Mesh] OR "Weight Loss"[Mesh] OR "Exercise"[Title/Abstract] OR "Physical activit*"[Title/Abstract] OR "Walking"[Title/Abstract] OR "Steps"[Title/Abstract] OR "Step count"[Title/Abstract] OR "Training"[Title/Abstract] OR "Aerobic"[Title/Abstract] OR "Rehabilitation"[Title/Abstract] OR "Lifestyle intervention"[Title/Abstract] OR "Weight loss"[Title/Abstract] OR "Weight management"[Title/Abstract])

#4 ("Randomized Controlled Trial"[Publication Type] OR "Randomized Controlled Trials as Topic"[Mesh] OR "Randomized controlled trial"[Title/Abstract] OR "Random allocation"[Title/Abstract] OR "RCT"[Title/Abstract] OR "Clinical Trial"[Publication Type])

#5 #1 AND #2 AND #3 AND #4

1. **Web of Science：**

search strategy:

#1 TS= ("Obstructive Sleep Apnea" OR "Sleep Apnea" OR "OSA" OR "Sleep Apnoea" OR "Sleep Disordered Breathing" OR "Sleep Hypopnea")

#2 TS= (Telemedicine OR "Mobile Application*" OR "Wearable*" OR "Internet-Based Intervention" OR "Digital health" OR mHealth OR eHealth OR Telehealth OR Smartphone* OR "Mobile app*" OR "Activity tracker*" OR Pedometer OR "Remote monitoring" OR "Web-based" OR "Online program*" OR Virtual)

#3 TS= (Exercise OR Sports OR "Physical Exertion" OR "Physical functional performance" OR "Life Style" OR "Diet, Reducing" OR "Weight Loss" OR "Physical activit*" OR Walking OR Steps OR "Step count" OR Training OR Aerobic OR Rehabilitation OR "Lifestyle intervention" OR "Weight management")

#4 TS= (trial OR randomly OR placebo OR randomised OR randomized OR "Clinical Trial" OR "Multicenter Study" OR "Randomized Controlled Trial" OR "Controlled Clinical Trial" OR "Random Allocation")

#5 #1 AND #2 AND #3 AND #4

1. **Embase：**

search strategy:

#1 ti,ab,kw= ("Obstructive Sleep Apnea" OR "Sleep Apnea" OR "OSA" OR "Sleep Apnoea" OR "Sleep Disordered Breathing" OR "Sleep Hypopnea")

#2 ti,ab,kw= (Telemedicine OR "Mobile Application*" OR "Wearable*" OR "Internet-Based Intervention" OR "Digital health" OR mHealth OR eHealth OR Telehealth OR Smartphone* OR "Mobile app*" OR "Activity tracker*" OR Pedometer OR "Remote monitoring" OR "Web-based" OR "Online program*" OR Virtual)

#3 ti,ab,kw= (Exercise OR Sports OR "Physical Exertion" OR "Physical functional performance" OR "Life Style" OR "Diet, Reducing" OR "Weight Loss" OR "Physical activit*" OR Walking OR Steps OR "Step count" OR Training OR Aerobic OR Rehabilitation OR "Lifestyle intervention" OR "Weight management")

#4 ti,ab,kw= (trial OR randomly OR placebo OR randomised OR randomized OR "Clinical Trial" OR "Multicenter Study" OR "Randomized Controlled Trial" OR "Controlled Clinical Trial" OR "Random Allocation")

#5 #1 AND #2 AND #3 AND #4

1. **CINAHL：**

search strategy:

#1 XB=("Obstructive Sleep Apnea" OR "Sleep Apnea" OR "OSA" OR "Sleep Apnoea" OR "Sleep Disordered Breathing" OR "Sleep Hypopnea")

#2 XB= (Telemedicine OR "Mobile Application*" OR "Wearable*" OR "Internet-Based Intervention" OR "Digital health" OR mHealth OR eHealth OR Telehealth OR Smartphone* OR "Mobile app*" OR "Activity tracker*" OR Pedometer OR "Remote monitoring" OR "Web-based" OR "Online program*" OR Virtual)

#3 XB=(Exercise OR Sports OR "Physical Exertion" OR "Physical functional performance" OR "Life Style" OR "Diet, Reducing" OR "Weight Loss" OR "Physical activit*" OR Walking OR Steps OR "Step count" OR Training OR Aerobic OR Rehabilitation OR "Lifestyle intervention" OR "Weight management")

#4 XB=(trial OR randomly OR placebo OR randomised OR randomized OR "Clinical Trial" OR "Multicenter Study" OR "Randomized Controlled Trial" OR "Controlled Clinical Trial" OR "Random Allocation")

#5 #1 AND #2 AND #3 AND #4

1. **Cochrane Library：**

search strategy:

#1 ti,ab,kw= ("Obstructive Sleep Apnea" OR "Sleep Apnea" OR "OSA" OR "Sleep Apnoea" OR "Sleep Disordered Breathing" OR "Sleep Hypopnea")

#2 ti,ab,kw= (Telemedicine OR "Mobile Application*" OR "Wearable*" OR "Internet-Based Intervention" OR "Digital health" OR mHealth OR eHealth OR Telehealth OR Smartphone* OR "Mobile app*" OR "Activity tracker*" OR Pedometer OR "Remote monitoring" OR "Web-based" OR "Online program*" OR Virtual)

#3 ti,ab,kw= (Exercise OR Sports OR "Physical Exertion" OR "Physical functional performance" OR "Life Style" OR "Diet, Reducing" OR "Weight Loss" OR "Physical activit*" OR Walking OR Steps OR "Step count" OR Training OR Aerobic OR Rehabilitation OR "Lifestyle intervention" OR "Weight management")

#4 ti,ab,kw= (trial OR randomly OR placebo OR randomised OR randomized OR "Clinical Trial" OR "Multicenter Study" OR "Randomized Controlled Trial" OR "Controlled Clinical Trial" OR "Random Allocation")

#5 #1 AND #2 AND #3 AND #4

1. **ClinicalTrials.gov：**

search strategy:

Condition or disease: "Obstructive Sleep Apnea" OR "Sleep Apnea" OR "OSA" OR "Sleep Apnoea" OR "Sleep Disordered Breathing" OR "Sleep Hypopnea"

Intervention/treatment: Telemedicine OR "Mobile Application*" OR "Wearable*" OR "Internet-Based Intervention" OR "Digital health" OR mHealth OR eHealth OR Telehealth OR Smartphone* OR "Mobile app*" OR "Activity tracker*" OR Pedometer OR "Remote monitoring" OR "Web-based" OR "Online program*" OR Virtual

Other terms: Exercise OR Sports OR "Physical Exertion" OR "Physical functional performance" OR "Life Style" OR "Diet, Reducing" OR "Weight Loss" OR "Physical activit*" OR Walking OR Steps OR "Step count" OR Training OR Aerobic OR Rehabilitation OR "Lifestyle intervention" OR "Weight management"
